# Copy number gains at chr3p25 and chr11p11 are associated with lymph node involvement and survival in muscle-invasive bladder tumors

**DOI:** 10.1371/journal.pone.0187975

**Published:** 2017-11-15

**Authors:** Karla J. Lindquist, Thomas Sanford, Terence W. Friedlander, Pamela L. Paris, Sima P. Porten

**Affiliations:** 1 Department of Urology, University of California San Francisco, San Francisco, CA, United States of America; 2 Department of Medicine, Division of Hematology & Medical Oncology, University of California San Francisco, San Francisco, CA, United States of America; Centro Nacional de Investigaciones Oncologicas, SPAIN

## Abstract

Patients with muscle-invasive bladder cancer (MIBC) have poorer prognoses if cancer has metastasized to the lymph nodes. Genomic markers of lymph node involvement (LNI) would be useful for treatment planning, especially if measured at the biopsy stage, but large-scale studies of tumor tissue at any stage are needed to discover robust markers of LNI. We performed a genome-wide query of copy number alterations (CNA) in 237 MIBC surgical tumor specimens from patients in The Cancer Genome Atlas who had radical cystectomy and lymphadenectomy without neoadjuvant treatment. Pathology reports were independently reviewed to confirm LNI, and copy number data was analyzed to confirm gene-level gains and losses while adjusting for tumor purity and ploidy. Using logistic regression and elastic net models, we identified the CNA most significantly associated with LNI. Multivariable logistic regression was used to describe these CNA associations while adjusting for clinical variables. Kaplan-Meier and Cox regression were used to describe their association with overall survival. Gains in 26 genes were identified as having strong associations with LNI. After adjusting for age, gender, race, pathological tumor stage, histology, and number of nodes examined, gains in 22 genes on chr3p25 or chr11p11 remained significantly associated with LNI (p<0.01) and improved model discrimination over clinical variables alone (p = 0.04). They were also associated with shorter overall survival (adjusted p = 0.02). These results suggest that a simple genomic test for gains in chr3p25 and chr11p11 could inform adjuvant treatment or clinical trial decisions if validated in external cohorts. Additional studies will also be needed to determine if these CNA are detectible in biopsy tissue and can inform clinical decisions at the preoperative stage.

## Introduction

Bladder cancer is the sixth most common cancer in the US. Metastatic bladder cancer is estimated to have caused 16,390 deaths in 2016 in the US, and almost all of these cases will have originated as muscle-invasive bladder cancer (MIBC) [1].

MIBC patients have a wide spectrum of clinical characteristics, but have markedly worse prognosis if cancer has metastasized with lymph node involvement (LNI). Though radical cystectomy with lymphadenectomy can improve outcomes, studies have shown that 25% of patients are clinically under-staged prior to cystectomy [2], and patients with LNI have higher recurrence and mortality rates compared to patients without LNI [3–5]. To illustrate this, 5-year overall and disease-free survival rates in patients with LNI are 35% and 31%, respectively, versus 89% and 69% for patients without LNI [3]. For patients with LNI, prognoses can vary, despite efforts to improve the TNM staging system to better reflect prognosis using lymph node information [6–11]. Better methods for determining prognosis in these patients are required to identify those at highest risk of recurrence who might benefit from the additional chemotherapy, radiotherapy, or a clinical trial.

Several RNA-based tests measuring gene expression in MIBC tumor samples taken at cystectomy have been associated with LNI [12–14] and outcomes [15–18], but are not yet in routine clinical use because they have not been validated in external cohorts or do not add significant value over clinical information alone. DNA-based markers, such as gene copy number alterations (CNA) that are associated with expression levels, may be more robust than RNA-based tests alone. The association of CNA in primary MIBC tumors and LNI has not previously been described. In this study, we aimed to discover robust genomic markers of LNI. We also tested for association of these markers with overall survival as a secondary outcome, and with expression and other biological relationships as forms of internal validation. We analyzed microarray-based copy number (CN) data from cystectomy-derived tumor samples from 237 MIBC patients represented in The Cancer Genome Atlas (TCGA) [19]. We independently reviewed pathology reports and re-processed raw CN data to confirm LNI and gene-level CNA in the tumor samples. Our goal was to test the hypothesis that there are CNA in MIBC tumor genomes that are associated with LNI and survival and that, along with other clinical and molecular markers, may be clinically useful in determining prognosis for MIBC patients.

## Materials and methods

### Patient and biological specimen selection

All sites contributing samples to TCGA obtained local IRB approval and informed consent prior to submission. The Human Research Protection Program of the Office of Ethics and Compliance at the University of California, San Francisco does not consider this study subject to IRB review because it is research conducted with de-identified or coded private information obtained from IRB-approved repositories that do not include any protected health identifiers.

In this retrospective study that follows REMARK criteria [20], we tested for CNA associated with LNI at the time of cystectomy and also short-term survival for up to two years post-cystectomy, since TCGA had follow-up data for this duration on all patients. TCGA selection criteria required that patients be diagnosed with MIBC (TNM stages T2-T4) with no prior malignancies or neoadjuvant treatment prior to cystectomy. Tumor and matched normal samples from peripheral blood or adjacent histologically normal tissue (>2cm from tumor) were immediately frozen after cystectomy and shipped to the Broad Institute for genomic testing by each treating institution. Samples were included only if >50% were urothelial carcinoma histology (versus variant histology such as squamous differentiation). Pathology review was carried out by four independent pathologists with training in genitourinary pathology. Additional information on patient selection, sample preservation, assay protocols, quality control, reproducibility have been reported previously [19].

For this study, it was required that pathology reports be available in cBioPortal [21–22] for our independent review, and that each patient underwent a radical cystectomy and lymphadenectomy, with a minimum of ten nodes examined, since it has been shown that patients with <10 nodes removed have significantly higher recurrence rates and shorter survival [23].

### Clinical and genomic measures considered

Two urologic oncologists (TS and SPP) performed a manual review of pathology reports in cBioPortal for each patient in the TCGA bladder cancer cohort to confirm pathological staging, tumor histology, and the number of involved and uninvolved nodes. Pathologic stage was classified according to the TNM classification of the American Joint Committee on Cancer (AJCC) cancer staging manual 7th edition [24]. We categorized histology as pure urothelial, urothelial with squamous differentiation, or other. Lymph node density was defined as the ratio of positive to total nodes examined. Extracapsular extension (ECE) was categorized as present or absent. Age at diagnosis, gender and race data were obtained from TCGA. Race was self-identified and categorized as white or non-white.

As previously reported [19], genomic assays for tumor and normal samples were conducted at the Broad Institute (BI). CN was measured on Affymetrix Genome-Wide Human 6.0 microarrays. The BI used circular binary segmentation (CBS) [25] in processing this data for making CNA calls. We independently processed the data—hereafter referred to as the University of California, San Francisco (UCSF) pipeline—using Allele-Specific Copy Number Analysis of Tumors (ASCAT) to adjust for tumor purity and ploidy [26]. CNA for each RefSeq gene [27] in chromosomes 1–22 and X were called by applying the Genomic Identification of Significant Targets in Cancer 2.0 (GISTIC2.0) algorithm [28] to both the BI and UCSF-processed data. The same GISTIC2.0 parameters were used for the BI and UCSF processing pipelines, with loss thresholds of log_2_(CN/2) < −0.1 and gain thresholds of log_2_(CN/2) > 0.1. The GISTIC2.0 algorithm empirically derives amplification and deletion thresholds. “BI-UCSF Calls” were created for each patient, categorized as concordant gains, losses, non-CNA, or discordant calls (S1 Fig). We also examined mRNA expression, somatic mutations, and methylation levels in tumor samples for the genes that had CNA significantly associated with LNI. Additional methodological details can be found in the S1 Text and on the TCGA website (http://cancergenome.nih.gov/).

### Statistical and biological analysis

For bivariable tests comparing clinical and pathological characteristics by LNI, two-sided Wilcoxon rank-sum tests [29] for continuous variables and χ^2^ tests for categorical variables were used. To identify genes with CNA associated with LNI, a unique analysis pipeline consisting of four main stages was developed (S1 Fig). The goal with the first two stages was to screen genes for their association with LNI for the final two stages. In the first stage, all 20,333 RefSeq genes were liberally screened for CNA-LNI associations using bivariable logistic regression models with a multiple comparison-corrected false discovery rate (FDR) q-value of <0.2. The purpose of this first stage was to make the next stage more computationally feasible while focusing on the genes most likely to have robust LNI associations. In the second more stringent screening stage, all genes filtered through the first stage were entered into a multivariable elastic net (EN) regression model [30]. The EN method was chosen because it can simultaneously handle a large number of variables (genes) relative to the number of observations (patients), and can aid in selecting the subset of variables, or groups of correlated variables, that have the strongest association with the outcome. We identified the optimal EN model parameters and most associated subset of genes by using 5-fold cross-validation to assess prediction error in separate samples from those used to fit the model. This process was repeated 100 times, and we selected genes that were the most associated in at least 95% of these replications.

In the third stage of analysis, genes filtered by EN models were grouped into sets based on genomic position and EN model coefficient similarity. In the fourth stage, gene sets were entered into multivariable logistic regression models to test for association with LNI, with and without adjusting for clinical covariates (age, gender, race, pathological tumor stage, histology, and the number of nodes examined). C-statistics for models containing the gene sets or clinical data alone versus all combined were compared using DeLong’s test [31]. Logistic regression model fit was assessed using the Hosmer-Lemeshow test [32], and accuracy was further assessed using plots of actual versus predicted probabilities of LNI.

For overall and disease-free survival analyses, we calculated the time in months from the date of cystectomy to the date of each event, 24 months, loss-to-follow-up, or death for disease-free analyses (whichever came first). We also calculated overall survival up to 12 months post-cystectomy. Log-rank statistics and Kaplan-Meier [33] curves were used to compare overall and disease-free survival by LNI and gene set CNA. Cox proportional hazards models [34] were used to adjust for clinical covariates, with proportional hazards assumptions tested on the basis of Schoenfeld residuals [35], and accuracy was assessed using plots of actual versus predicted survival times.

For biological analyses, the Molecular Signatures Database (MSigDB) [36] was used to determine whether the final gene list had significant overlap with any functional gene sets curated from online pathway databases and PubMed. An FDR q-value of 0.05 was selected as the threshold for significance. Wilcoxon rank-sum tests were used to test for differences in average tumor expression and methylation levels by gene set CNA. cBioPortal was used for visualizing other alterations including amplifications and deletions and somatic mutations by LNI, and for performing network analysis to identify functionally related genes and drug interactions. Finally, other cancer types with the same CNA were searched for in both TCGA and the Catalog of Somatic Mutations in Cancer (COSMIC) [37].

All statistical tests were two-sided, and all 95% confidence intervals (CI) and p-values were estimated by 1,000 bootstrap repetitions. Analyses were conducted with R software version 3.3 [38] or Stata version 11.2 [39]. See S1 Text for additional details.

## Results

### Patient and biological specimens

There were 412 patients in the TCGA bladder cancer cohort with MIBC. We excluded one patient without a pathology report, 111 patients for whom pathology data was insufficient for LNI verification, three for whom CN data was unavailable, and 60 patients with less than ten nodes examined. This left 237 patients for study, all of whom were diagnosed between 1999 and 2013. One-hundred patients (42%) had LNI and 137 patients (58%) did not. Age at diagnosis, gender, race, tumor histology, and the number of nodes examined did not differ by LNI, but LNI patients were significantly more likely to have higher pathological tumor stages (p<0.01) (Table 1).

**Table 1:** Patient and clinical characteristics by lymph node involvement.

Characteristic,	Lymph Node Involvement		P-value^a^
Median (Range) or %	No (N = 137)	Yes (N = 100)	
Age at diagnosis	69 (43–88)	69 (45–90)	0.19
Gender: Female	28%	27%	0.82
Race: Non-white	14%	6%	0.08
Pathological Tumor Stage: T2	34%	15%	<0.01
T3	57%	62%	
T4	9%	23%	
Histology: Pure urothelial	67%	66%	0.07
Squamous	17%	9%	
Other	16%	25%	
Number of nodes examined	24 (10–141)	23 (10–170)	0.57
Pathological Node Stage: N0	100%	-	-
N1	-	34%	
N2	-	59%	
N3	-	7%	
Extracapsular nodal extension^b^	-	62%	-
Lymph node density (%)	-	10 (1–23)	-

^a^P-value by χ2 for categorical variables, Wilcoxon rank-sum for continuous variables.

^b^Missing in 29% of patients with lymph node involvement.

Median overall survival time was 14.3 months for the 109 patients (46%) who died at any time during follow-up, and 24.0 months for those who were censored. Within one year after cystectomy 44 (19%) had died, and within two years 88 (37%) had died. Recurrence could not be determined for 55 patients, leaving 182 patients for disease-free survival analyses. Median disease-free survival time was 19.0 months for the 84 patients (46%) who recurred at any time, and 39.2 months for those who were censored. Within two years after cystectomy 19 (10%) had recurred.

### Copy number associations

In the first analysis stage to liberally screen for genes associated with LNI, bivariable logistic regression models identified 8,794 of 20,333 RefSeq genes with CNA (based on BI-UCSF calls) that were associated with LN positivity at the q<0.2 level. After a second more stringent screening with multivariable EN models, 100 of these genes were associated with LNI. When these two stages were repeated using the BI and UCSF calls separately, 26 of these 100 genes were significantly associated with LNI. The 26 genes were therefore considered to have the most robust associations, and were retained for further analyses. They were then grouped into six sets based on genomic proximity and similarity in the degree of LNI associations. Twelve contiguous genes in a 0.3Mb section of chromosome 3, cytoband p25.3 (hg19 chr3:9691117–9958084) comprised one set (the “chr3p25” set), ten contiguous genes in a 2.7Mb section of chromosome 11, cytoband p11.2 (chr11:43702143–46402104) comprised another set (the “chr11p11” set), and the remaining four genes comprised single-gene sets on other chromosomes/chromosomal arms (*EPHA3*, *ATE1*, *HIPK3*, *AATF*). Odds ratios (OR) from bivariable analyses of each of the individual genes and gene sets are shown in Table 2.

**Table 2:** Bivariable logistic regression results for the 26 genes and 6 gene sets.

Gene	Description	Cytoband	CNA	OR^a^	Gene Set	OR^a^
*MTMR14*	myotubularin related protein14	chr3p25	Gain	2.95	chr3p25	2.95
*CPNE9*	copine family member IX	chr3p25	Gain	2.95		
*BRPF1*^*b*^	bromodomain and PHD finger 1	chr3p25	Gain	2.95		
*OGG1*	8-oxoguanine DNA glycosylase	chr3p25	Gain	2.95		
*CAMK1*^*b*^	calcium/calmodulin-dependent protein kinase I	chr3p25	Gain	2.95		
*TADA3*	transcriptional adaptor 3	chr3p25	Gain	2.95		
*ARPC4*	actin related protein 2/3 complex, subunit 4, 20kDa	chr3p25	Gain	2.95		
*TTLL3*	tubulin tyrosine ligase-like family, member 3	chr3p25	Gain	2.95		
*RPUSD3*	RNA pseudouridylate synthase domain containing 3	chr3p25	Gain	2.95		
*CIDEC*	cell death-inducing DFFA-like effector c	chr3p25	Gain	2.95		
*JAGN1*	jagunal homolog 1	chr3p25	Gain	2.95		
*IL17RE*	interleukin 17 receptor E	chr3p25	Gain	2.95		
*HSD17B12*	hydroxysteroid (17-beta) dehydrogenase 12	chr11p11	Gain	4.36	chr11p11	3.78
*ALKBH3*	alkylation repair homolog 3	chr11p11	Gain	4.36		
*C11orf96*^*b*^	chr 11 open reading frame 96	chr11p11	Gain	4.36		
*ACCSL*	*ACCS*-like	chr11p11	Gain	4.36		
*ACCS*	1-aminocyclopropane-1-carboxylate synthase homolog	chr11p11	Gain	4.36		
*EXT2*^*b*^	exostosin 2	chr11p11	Gain	4.36		
*ALX4*	ALX homeobox 4	chr11p11	Gain	4.36		
*PHF21A*^*b*^	PHD finger protein 21A	chr11p11	Gain	4.29		
*CREB3L1*	cAMP responsive element binding protein 3-like 1	chr11p11	Gain	4.29		
*DGKZ*^*b*^	diacylglycerol kinase, zeta	chr11p11	Gain	4.29		
*EPHA3*	EPH receptor A3	chr3p11	Gain	3.29	*EPHA3*	3.29
*ATE1*	arginyltransferase 1	chr10q26	Gain	4.47	*ATE1*	4.47
*HIPK3*^*b*^	homeodomain interacting protein kinase 3	chr11p13	Gain	3.38	*HIPK3*	3.38
*AATF*	apoptosis antagonizing transcription factor	chr17q12	Loss	2.35	*AATF*	2.35

^a^OR = odds ratio from bivariable logistic regression for gene set gain (vs. no gain) or loss (vs. no loss). All are associated at p<0.01 except for *AATF* (p = 0.03).

^b^These genes significantly overlap a *TP53* target gene set (q<0.01).

For the final analyses, the chr3p25 and chr11p11 gene sets were combined since gains often co-occurred (χ^2^ p = 0.07) and were more strongly associated with LNI together (OR = 3.85, 95% CI 2.19–6.78, Table 3). Overall, 86 patients (36.3%) had gains in the chr3p25 or chr11p11 genes, of which 54 (62.8%) were LN-positive. Of the 151 patients without gains in these genes, 46 (30.5%) were LN-positive (χ^2^ p<0.01). In a multivariable logistic regression model including the combined chr3p25, chr11p11 and four single-gene sets, the single-gene sets were no longer associated with LNI. Since these four genes are also in different genomic regions than the rest, they were excluded from further analyses. After adjusting for clinical covariates (age, gender, race, pathological tumor stage, histology, and the number of nodes examined), the chr3p25, chr11p11 gene set remained significantly associated (p<0.01) with LN-positivity (OR = 3.76, 95% CI 1.96–7.23, Table 3). Model discrimination measured by c-statistics was significantly improved when including the gene sets with clinical data, compared to a model with clinical data alone (c = 0.744 versus c = 0.691 respectively, p = 0.04). The overall fit of the multivariable model with gene sets and clinical variables was good according to the Hosmer-Lemeshow test and predicted versus actual LNI (S2 Fig).

**Table 3:** Logistic regression results describing gene set association with lymph node involvement, with and without adjusting for clinical variables.

Variable		OR^a^	95% CI^b^	P-value
		Unadjusted		
Gene set chr3p25, chr11p11	Gains vs. no gains	3.85	2.19–6.78	<0.01
		Adjusted		
Gene set chr3p25, chr11p11	Gains vs. no gains	3.76	1.96–7.23	<0.01
Years of age at diagnosis		1.02	0.99–1.06	0.19
Gender:	Female vs. male	1.15	0.58–2.26	0.70
Race:	Non-white vs. white	0.95	0.28–3.21	0.28
Pathological tumor stage:^c^	T2 vs. T3 vs. T4	2.43	1.36–4.32	<0.01
Histology:	Squamous vs. pure urothelial	0.60	0.21–1.70	0.32
	Other vs. pure urothelial	1.58	0.77–3.26	0.20
Number of nodes examined		1.01	0.99–1.02	0.36

^a^OR = odds ratio for lymph node involvement.

^b^CI = confidence interval, bootstrapped (1,000 repetitions).

^c^Treated as an ordinal variable since there was a linear increase in log odds of lymph node involvement with increasing stages.

**Fig 1:**
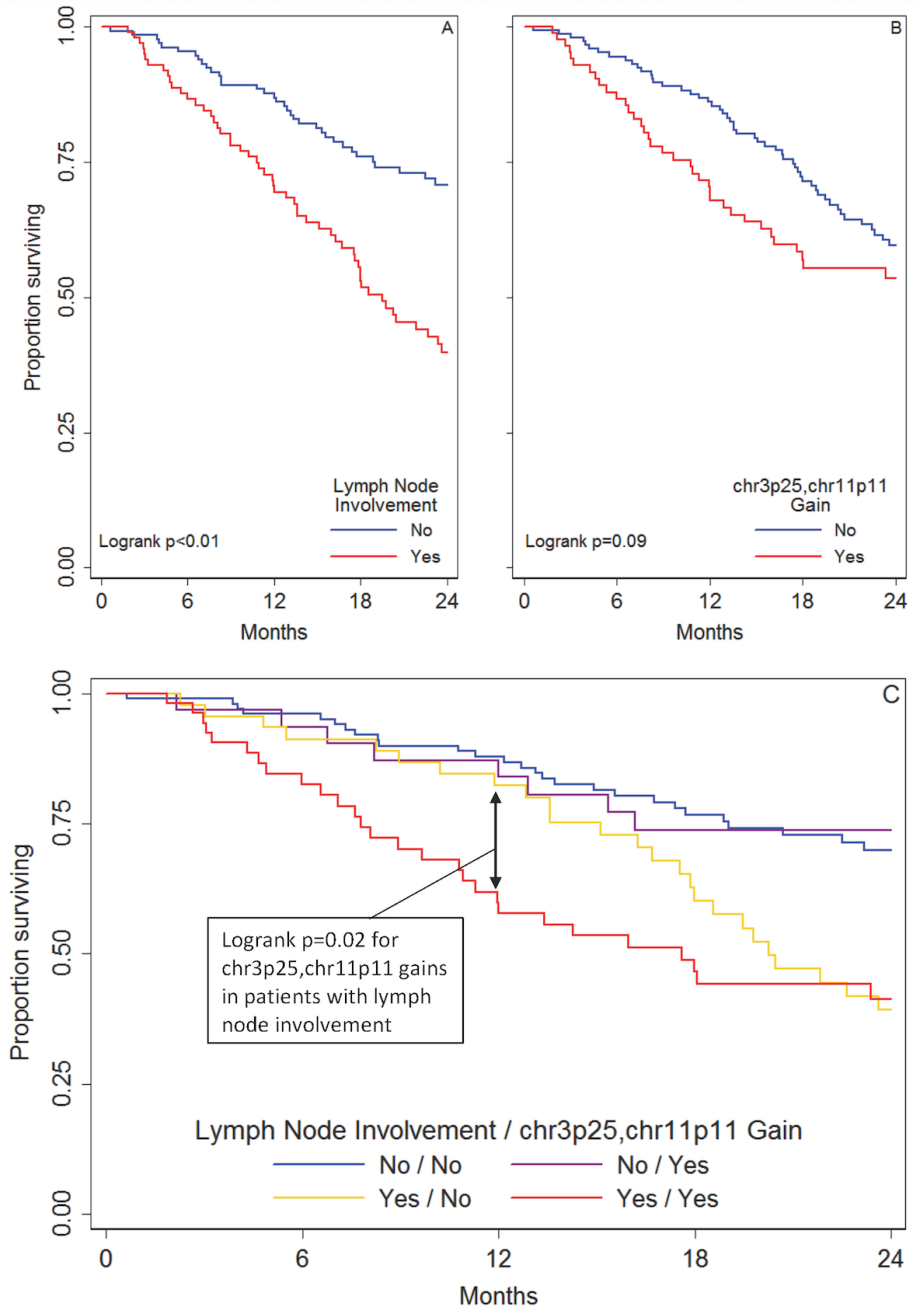
Kaplan-Meier survival curves for 24-month survival. A. By lymph node involvement, B. By chr3p25, chr11p11 gain. C. By lymph node involvement and chr3p25, chr11p11 gain.

In Kaplan-Meier analyses, LNI was significantly associated with two-year overall survival (p<0.01), but gains in the chr3p25, chr11p11 gene set were not associated with two-year overall survival (p = 0.09) (Fig 1). However, both LNI and gains in the chr3p25, chr11p11 gene set were significantly associated with one-year overall survival (p<0.01 for both). Stratified Kaplan-Meier curves revealed that the association between chr3p25, chr11p11 and one-year survival was only significant in patients with LNI (p = 0.02), not in those without LNI (p = 0.89) (Fig 1). Neither LNI nor chr3p25, chr11p11 were associated with two-year disease-free survival.

In Cox models adjusting for age, gender, race, pathological tumor stage, histology and number of nodes examined, chr3p25, chr11p11 gains were not associated with two-year overall survival, but they were associated with one-year survival (HR = 2.20, 95% CI 1.11–4.36). LNI and chr3p25, chr11p11 gains did not have a significant interaction term (p = 0.12) in the multivariable model for one-year survival, but this model revealed that patients with LNI had significantly shorter survival times associated with gains in chr3p25, chr11p11 (HR = 2.66, 95% CI 2.09–3.46), and patients without LNI did not (HR = 1.04, 95% CI 0.80–1.48). Proportional hazards assumptions were met in this Cox model, and predictions were accurate over time patients with and without LNI (S3 Fig).

### Biological analyses

MSigDB (36) was used to test the 26 genes for significant overlap with functional gene sets. A previously defined *TP53* target gene set [40] significantly overlapped the 26 genes (FDR q<0.01, Table 2). Two of the seven overlapping *TP53* target genes were in chr3p25, four were in chr11p11, and one (*HIPK3*) was in chr11p13. The combined chr3p25, chr11p11 set contained six *TP53* target genes and 16 non-*TP53* target genes.

Using mRNA measurements, expression levels were higher in tumors with gains versus no gains in the chr3p25 genes (difference in median z-scores = 0.87, Wilcoxon rank-sum p<0.01) and in the chr11p11 genes (difference in median z-scores = 0.34, Wilcoxon rank-sum p<0.01) (S4 Fig). We also tested for associations between LNI and expression in 27 genes from other regions of the genome described by Seiler et al. [13] as part of their expression-based signature of LNI. Adjusting the multivariable logistic regression model for mRNA levels in the Seiler genes did not change the significance of the association between gains in the chr3p25, chr11p11 set and LNI (p<0.01).

Lower methylation levels were associated with gains in chr3p25 (p<0.01), but adjusting for this also had no impact on the CNA associations with LNI. Mutations in the 26 genes were uncommon and not associated with CNA or LNI (S5 Fig). However, *TP53* mutations were more likely to occur in patients with gains versus no gains in the chr3p25, chr11p11 set (50% vs. 28% respectively, χ^2^ p = 0.03), but were not associated with LNI.

In a network analysis of the 26 genes performed using cBioPortal [21–22], ten were functionally connected to other genes via state change or expression control, being in the same complex, or by drug interactions (S6 Fig). Five genes in this network (*E2F1*, *E2F3*, *EGFR*, *RAF1*, and *RB1*) significantly overlapped genes in the bladder cancer pathway from the Kyoto Encyclopedia of Genes and Genomes (KEGG) [41–42] database (FDR q<0.01). Six genes in the network are oncogenes (*AKT2*, *EGFR*, *EPHA3*, *PIK3CA*, *RAF1*, *TRIO*) and one is a tumor suppressor (*RB1*). Two of the 22 genes in chr3p25 or chr11p11 are targets of experimental small molecule therapeutics (DrugBank number DB03214 targeting *ACCS*, and DB08235 and DB08236 targeting *ARPC4*).

CNA in the same direction in our 26 genes were noted in TCGA or COSMIC for bone, breast, intestine, ovary, prostate, S1 Table).

### Sensitivity analyses

Genome-wide CNA occurred in similar regions for BI and UCSF processing pipelines (S7 Fig). Cohen’s kappa statistics [43] indicated significant agreement in the direction of CNA calls for 97% of all genes (FDR q<0.05). The median proportion of RefSeq genes with perfect agreement was 69%, which was not significantly associated with ASCAT-estimated tumor ploidy (median 3.0, range 1.5–5.3) or purity (median 60%, range 23–100%), or with LNI. Bivariable logistic regression results were compared for all RefSeq genes using the BI versus the UCSF calls. Coefficients and–log_10_(p-values) were positively correlated (p<0.01), although UCSF ASCAT-based calls produced more conservative results as expected (median gain/loss OR = 1.57 for BI versus OR = 1.22 for UCSF).

No genes had discordant BI versus UCSF calls associated with LNI. Adding discordant calls to the reference group (rather than just concordant non-CNA calls) resulted in the same 26 genes with CNA associations, plus an additional two genes that are in the chr11p11 region (*API5* and *TTC17*, both having gains associated with LNI).

In multivariable models adjusting for age, gender, race, pathological tumor stage, histology, and number of nodes examined, amplifications (extreme gains of log_2_(CN/2)>0.9), empirically determined by GISTIC2.0) in the chr3p25 or chr11p11 genes were significantly associated with LNI (OR = 4.08, 95% CI 1.44–11.57). However, only 27 patients (11%) had amplifications in these genes.

## Discussion

We have identified genes in chr3p25 and chr11p11 with CN gains that are associated with LNI and survival in patients with MIBC. These associations remained significant after adjusting for clinical characteristics, and discrimination between patients with and without LNI was significantly improved over clinical variables alone. To our knowledge this is the first study to describe tumor-based CN associations with LNI in MIBC patients.

After identifying the genes with CNA that were most associated with LNI, we used various methods to understand the biological mechanisms behind these associations. Over a quarter of the genes in our final list of 26 genes significantly overlapped a *TP53* target gene set [40], suggesting that these could be the drivers of the associations we have observed. Network analyses revealed that some of the other genes may also have biological relevance. A total of nine chr3p25 and chr11p11 genes were functionally connected to genes in the KEGG bladder cancer pathway (*E2F1*, *E2F3*, *EGFR*, *RAF1*, *RB1*) or to other cancer-related genes (*AKT2*, *PIK3CA*, *RB1*, *TRIO*). The oncogenes *RAF1* and *PPARG* are located in chr3p25.2, about 2.5Mb upstream from our genes in chr3p25.3. Gains in *RAF1* and *PPARG* occurred in 45% and 46% of patients, respectively (these were the same patients except for two with gains in only *PPARG*). These gains were significantly associated (p<0.05) with LNI in bivariable analyses, but did not remain associated in multivariable analyses including the genes we identified. The fact that higher expression, lower methylation levels, and more *TP53* gene mutations were all associated with gains in chr3p25 and chr11p11 also supports the biological relevance of our findings.

In addition to reviewing pathology reports, we independently processed CN data using a different method than what the BI used, since different methods can affect results. The BI used CBS [25] segmentation and did not account for tumor ploidy or purity. We used ASCAT [26] which adjusts copy number levels for these factors. Differences between the results were likely due to the fact that over half of the tumors in this cohort were estimated to be non-diploid and/or containing a mixture of tumor and normal cells. By using the CNA calls that were associated with LNI under both CBS and ASCAT, we may have excluded some associations that only one method captured, but are confident that our consensus system has resulted in associations that are robust to measurement bias and are therefore unlikely to be false positive findings. We also used cross-validation and reproducibility testing during our screening for CNA associations in order to reduce the risk of overfitting and internally validate our findings.

Another strength of our study is that our analyses were not confined to the regions of the genome with the most frequent or high-amplitude CNA. We considered all gains and losses as potentially associated with LNI. In fact, many of the frequently altered genes were not associated with LNI, including *CDKN2A* and others noted in the TCGA Research Network bladder cancer study [19]. Amplifications (extreme gains according to the GISTIC2.0 algorithm) [28] in chr3p25 and chr11p11 were strongly associated with LNI in this study, but the LN associations were primarily driven by lower-amplitude changes. In most patients, the gains in chr3p25 and chr11p11 were covered by just one or two segments of distinct copy number levels, so we believe these are the minimal regions associated with LNI. However, it is possible that more specific regions could be identified with different algorithms. All CNA that we identified were robust to two different CN processing pipelines and were detected using the reliable and stable DNA-based Affymetrix Genome-Wide Human Array 6.0 for measuring CN levels.

Although we have identified CNA in biologically plausible genes that have strong statistical associations with LNI and survival, our study does have some limitations. Despite our internal validations through pathology report reviews and statistical cross-validation, this does not guarantee successful validation in external cohorts. In addition, the gene sets were not associated with two-year disease-free survival (nor was LNI). This may be due to the fact that this study was underpowered to detect this because of incomplete overall survival and treatment information after cystectomy in the TCGA data. This also limited our ability to study overall survival beyond two years post-cystectomy, which would have been heavily censored and confounded. Both overall and disease-free survival should be further evaluated in appropriately powered prospective studies with more complete post-cystectomy clinical data.

We used cystectomy tissue for our analyses, whereas biopsy specimens (TURBT) prior to cystectomy are used for clinical staging and for pre-surgical treatment planning. Previous studies have compared classifiers and validated genomic signatures between different tissue sources, with the assumption that in the absence of neoadjuvant chemotherapy, the concordance of TURBT and cystectomy tissue may be high [14,16]. No direct comparisons between TURBT and cystectomy tissues in the literature in the same patient have been made. This is another limitation of our study. Only if validated in a prospective cohort using biopsy tissue from TURBT specimens could these biomarkers be associated with LNI prior to surgical extirpation and thereby provide guidance for neoadjuvant based treatments.

Using cystectomy-derived tumor samples, we detected CN gains in chr3p25 and chr11p11 genes that are associated with LNI and one-year survival over clinical measures alone. Prospective validation studies in external cohorts will be required to determine if a simple genomic test for these CNA can be used to identify patients with LNI that would not have been detected otherwise.

## Supporting information

S1 Fig Analysis pipeline used for identifying the 26 genes considered in final multivariable models. BI = Broad Institute, UCSF = University of California, San Francisco. CBS = circular binary segmentation. ASCAT = Allele-specific copy number analysis of tumors. CNA = copy number alteration. Mb = Megabase, SD = standard deviation.
(PDF)

S2 Fig Accuracy of multivariable logistic regression model predicting LNI with the combined gene set chr3p25, chr11p11 and clinical variables (age, gender, race, pathological tumor stage and race, and number of lymph nodes examined). 95% confidence intervals for the proportions are bootstrapped with 1,000 repetitions. Points are labeled with the number of LN-positive cases / total cases in the predicted probability group. The green line indicates perfect agreement between actual proportion of LN-positive cases and the predicted probability midpoint.
(PDF)

S3 Fig Accuracy of multivariable Cox regression model predicting one-year survival with the combined chr3p25, chr11p11 gene set, adjusted for clinical variables (age, gender, race, pathological tumor stage and race, and number of lymph nodes examined). A. For LN-negative patients. B. For LN-positive patients.
(PDF)

S4 Fig Normalized mRNA expression levels in tumor samples by copy number change in each of the 6 gene sets. Expression is expected to be higher in genes/sets with copy number gains, and lower in genes/sets with losses. Wilcoxon rank-sum tests were two-sided.
(PDF)

S5 Fig Copy number alterations (amplifications, deletions, gains and losses), differential expression, and somatic mutations in the 26 genes considered in final multivariable models, by lymph node involvement. (PDF)

S6 Fig Biological network analysis of the 26 genes with gains or losses associated with lymph node-positivity. Performed using cBioPortal. Alterations include copy number, differential expression, or somatic mutations in the TCGA bladder cancer cohort. Functional connections are via state change or expression control, being in the same protein complex, or having drug interactions.
(PDF)

S7 Fig Gain (red) and loss (blue) peaks across the genome, as determined by Genomic Identification of Significant Targets in Cancer (GISTIC2.0) for copy number levels from the Broad Institute (BI) and our (UCSF) copy number processing pipelines. The amplitude of each peak (y-axis for panels B and C) reflects both the magnitude and frequency of gains and losses across all tumor samples from the 237 patients in the cohort. The same GISTIC parameters were used to determine peaks for both processing pipelines. A. Chromosome. B. GISTIC peaks based on the BI pipeline data. C. GISTIC peaks based on the UCSF pipeline data.
(PDF)

S1 Table Tumors in The Cancer Genome Atlas (TCGA) and other studies in the Catalog of Somatic Mutations In Cancer (COSMIC version 78, 37) with the same copy number alterations in the 26 genes associated with lymph node-positivity in the TCGA bladder cancer cohort. (DOCX)

S1 Text Supplemental methods and references. (DOCX)
