# Supplementary figures and images for: Copy number gains at chr3p25 and chr11p11 are associated with lymph node involvement and survival in muscle-invasive bladder tumors
Source: PLoS One. 2017 Nov 15;12(11):e0187975. doi: 10.1371/journal.pone.0187975 (PMC5687759; doi:10.1371/journal.pone.0187975)

S1 Fig.

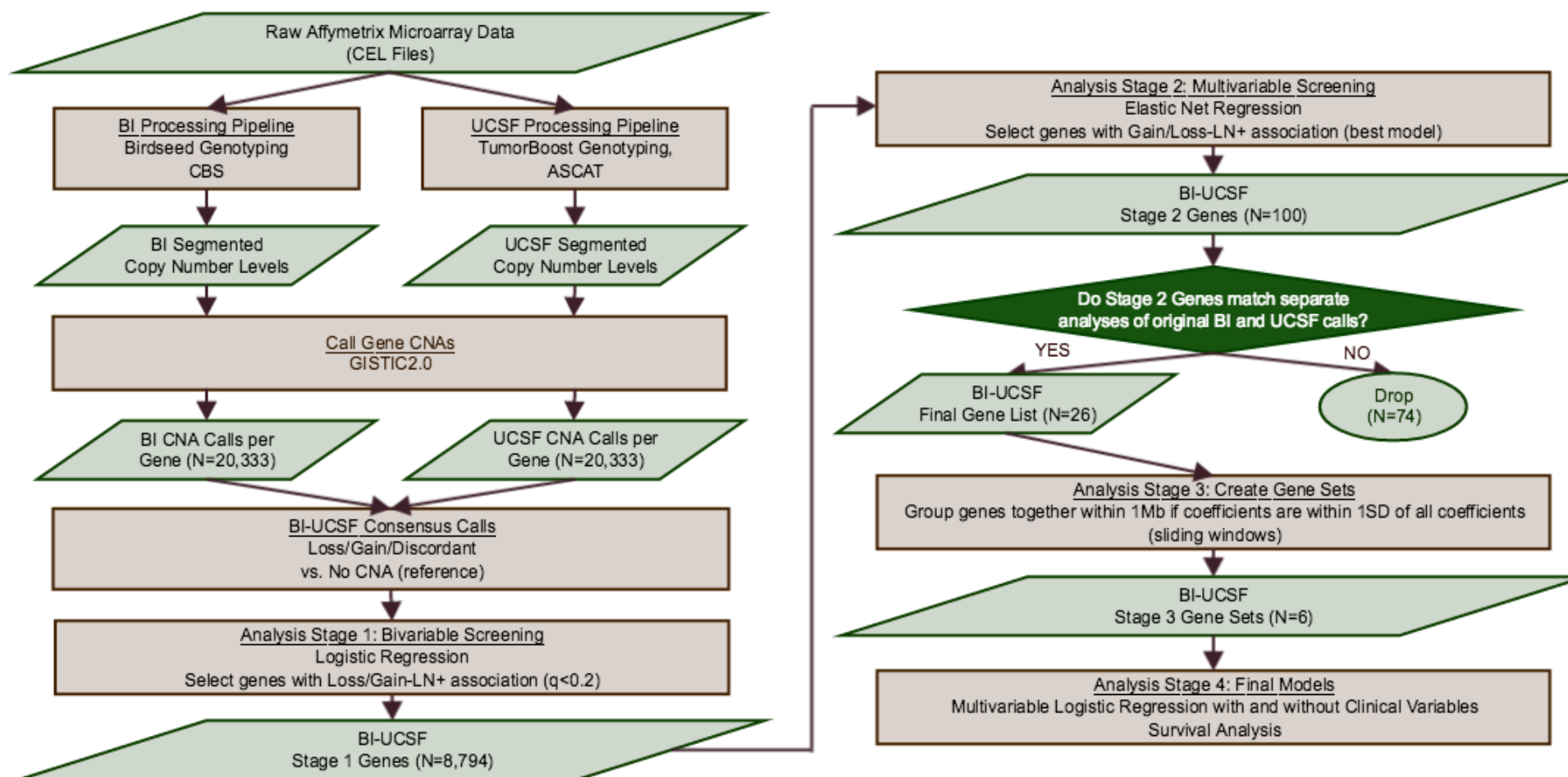

Supplement: S1 Fig — BI = Broad Institute, UCSF = University of California, San Francisco. CBS = circular binary segmentation. ASCAT = Allele-specific copy number analysis of tumors. CNA = copy number alteration. Mb = Megabase, SD = standard deviation. (PDF) [file pone.0187975.s001.pdf]

S2 Fig.

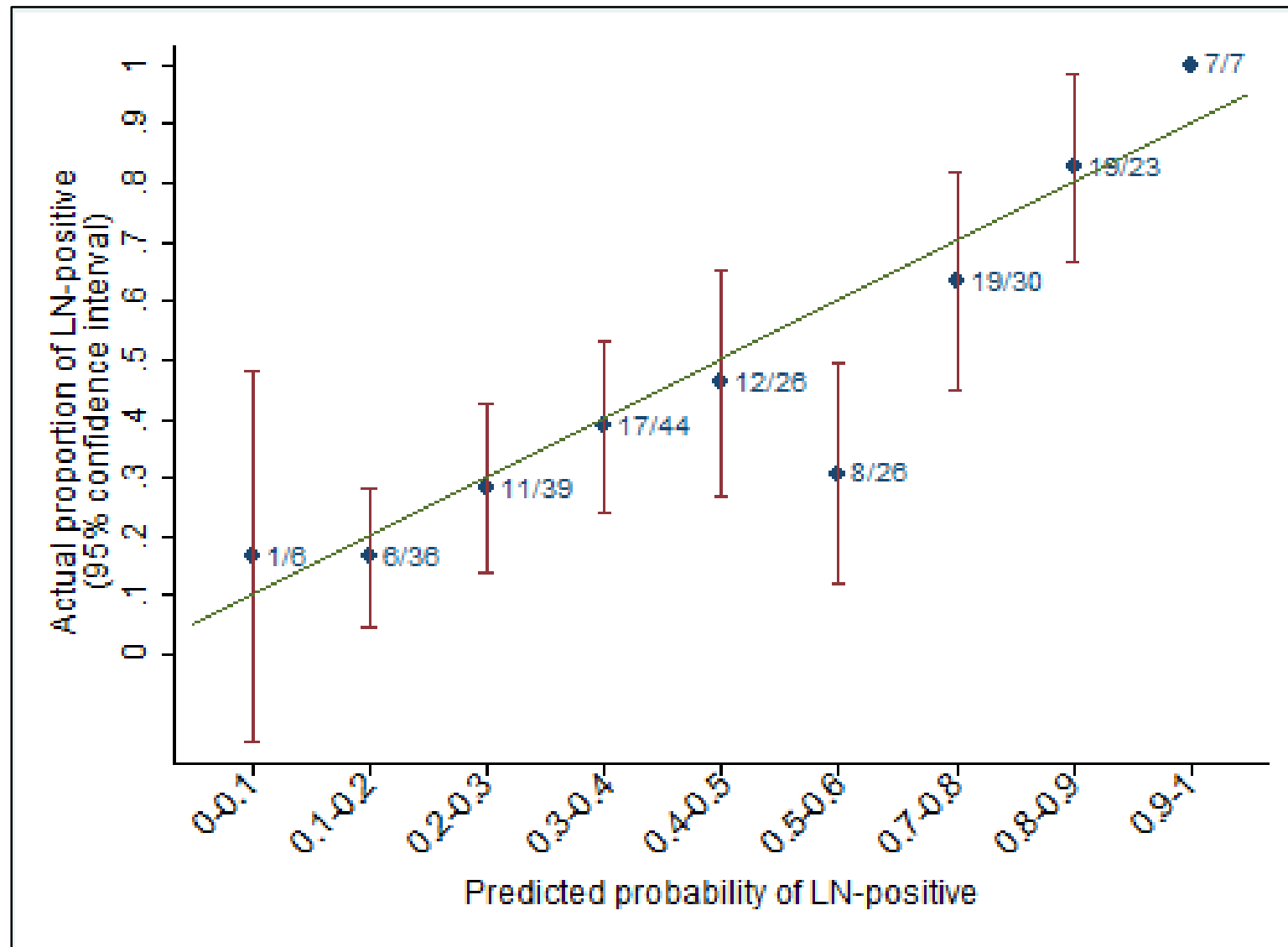

Supplement: S2 Fig — 95% confidence intervals for the proportions are bootstrapped with 1,000 repetitions. Points are labeled with the number of LN-positive cases / total cases in the predicted probability group. The green line indicates perfect agreement between actual proportion of LN-positive cases and the predicted probability midpoint. (PDF) [file pone.0187975.s002.pdf]

S3 Fig.

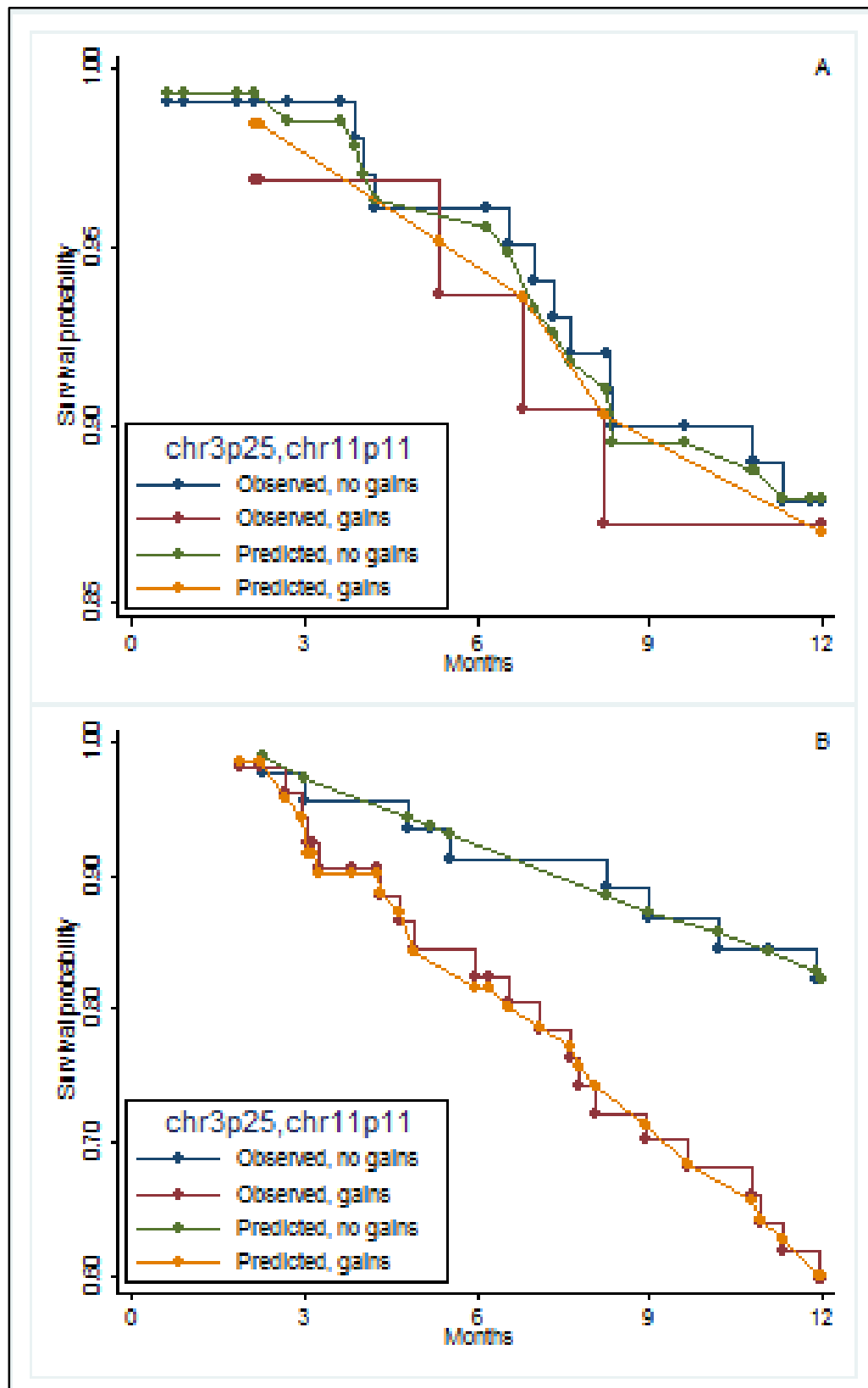

Supplement: S3 Fig — A. For LN-negative patients. B. For LN-positive patients. (PDF) [file pone.0187975.s003.pdf]

S4 Fig.

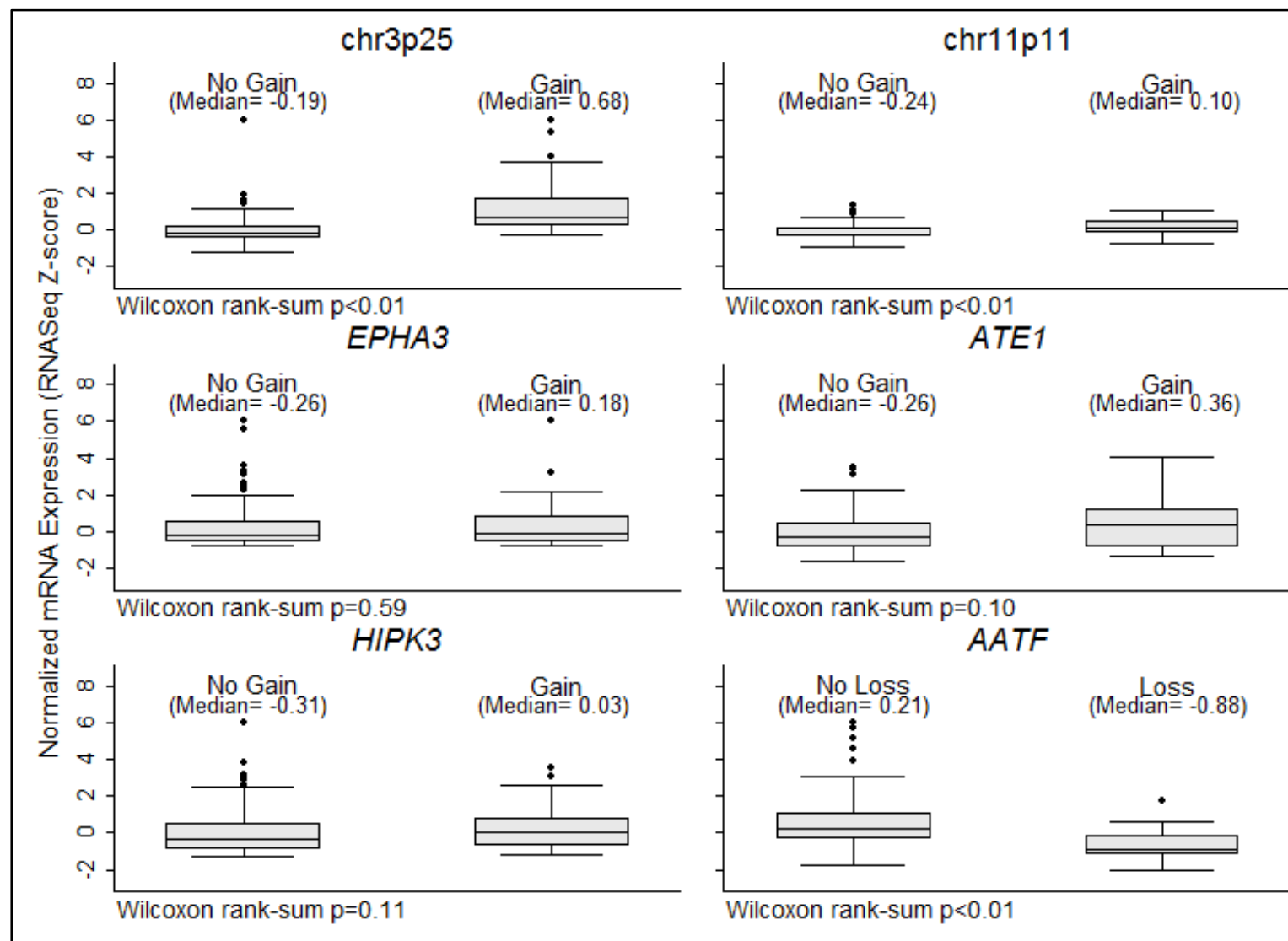

Supplement: S4 Fig — Expression is expected to be higher in genes/sets with copy number gains, and lower in genes/sets with losses. Wilcoxon rank-sum tests were two-sided. (PDF) [file pone.0187975.s004.pdf]

S6 Fig.

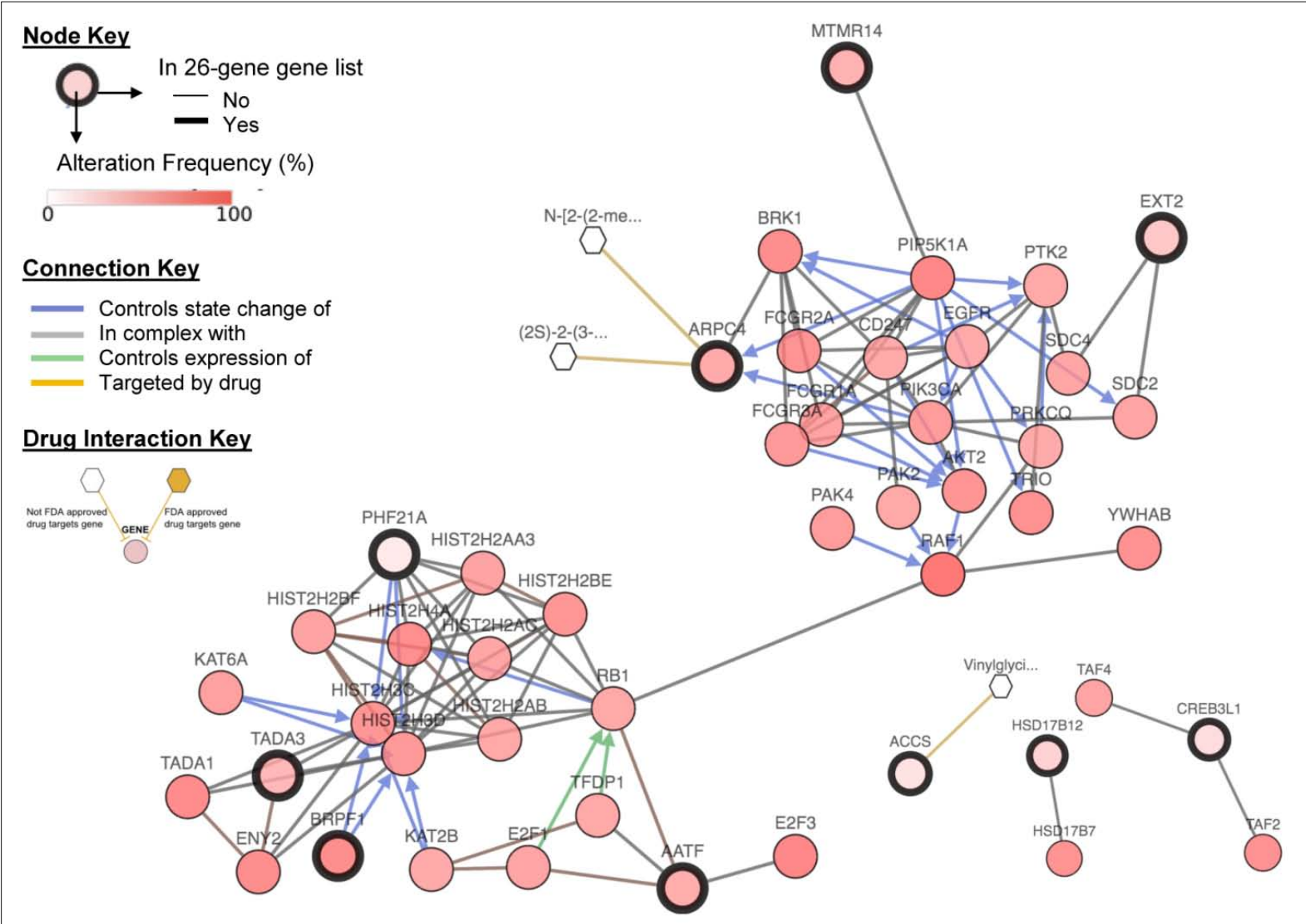

Supplement: S6 Fig — Performed using cBioPortal. Alterations include copy number, differential expression, or somatic mutations in the TCGA bladder cancer cohort. Functional connections are via state change or expression control, being in the same protein complex, or having drug interactions. (PDF) [file pone.0187975.s006.pdf]

S7 Fig.

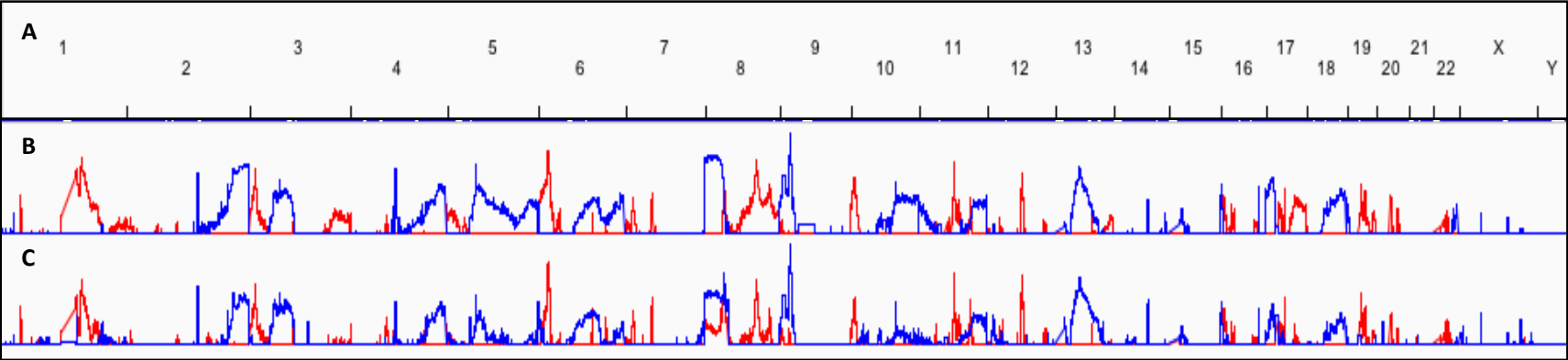

Supplement: S7 Fig — The amplitude of each peak (y-axis for panels B and C) reflects both the magnitude and frequency of gains and losses across all tumor samples from the 237 patients in the cohort. The same GISTIC parameters were used to determine peaks for both processing pipelines. A. Chromosome. B. GISTIC peaks based on the BI pipeline data. C. GISTIC peaks based on the UCSF pipeline data. (PDF) [file pone.0187975.s007.pdf]
