# Supplementary material for: Copy number gains at chr3p25 and chr11p11 are associated with lymph node involvement and survival in muscle-invasive bladder tumors
Source: PLoS One. 2017 Nov 15;12(11):e0187975. doi: 10.1371/journal.pone.0187975 (PMC5687759; doi:10.1371/journal.pone.0187975)
Supplement: S5 Fig — (PDF) [file pone.0187975.s005.pdf]

S5 Fig.

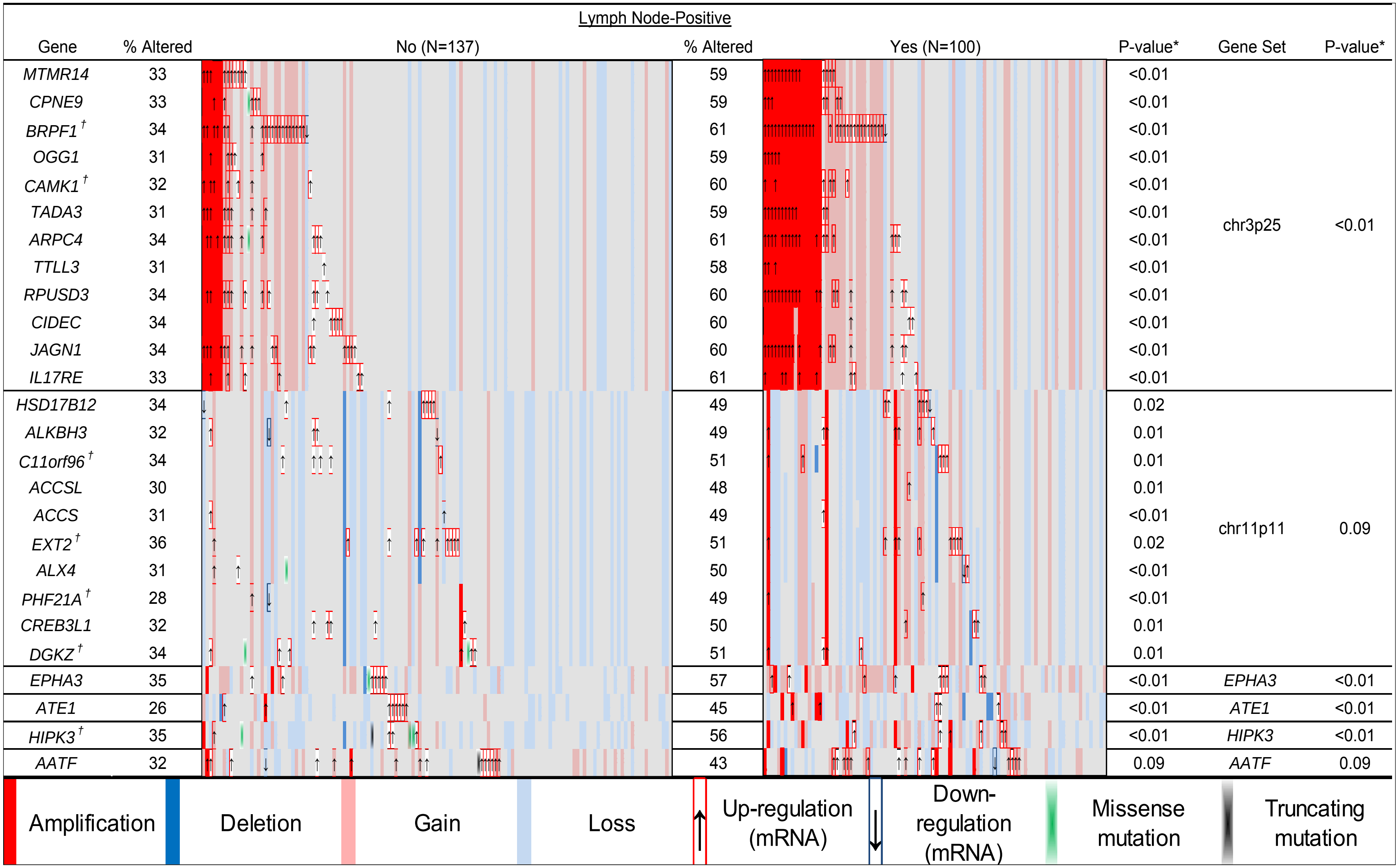

Gene	No (N=137) % Altered	Yes (N=100) % Altered	P-value*	Gene Set	P-value*
MTMR14	33	59	<0.01	chr3p25	<0.01
CPNE9	33	59	<0.01		
BRPF1†	34	61	<0.01		
OGG1	31	59	<0.01		
CAMK1†	32	60	<0.01		
TADA3	31	59	<0.01		
ARPC4	34	61	<0.01		
TTLL3	31	58	<0.01		
RPUSD3	34	60	<0.01		
CIDEC	34	60	<0.01		
JAGN1	34	60	<0.01		
IL17RE	33	61	<0.01		
HSD17B12	34	49	0.02	chr11p11	0.09
ALKBH3	32	49	0.01		
C11orf96†	34	51	0.01		
ACCSL	30	48	0.01		
ACCS	31	49	<0.01		
EXT2†	36	51	0.02		
ALX4	31	50	<0.01		
PHF21A†	28	49	<0.01		
CREB3L1	32	50	0.01		
DGKZ†	34	51	0.01		
EPHA3	35	57	<0.01	*EPHA3*	<0.01
ATE1	26	45	<0.01	*ATE1*	<0.01
HIPK3†	35	56	<0.01	*HIPK3*	<0.01
AATF	32	43	0.09	*AATF*	0.09

Legend: Amplification; Deletion; Gain; Loss; Up-regulation (mRNA); Down-regulation (mRNA); Missense mutation; Truncating mutation

*P-value by χ^2 comparing percent of genes with differential copy number, expression, or mutation, by lymph node status.
†These genes significantly overlap *TP53* target genes (q<0.01).
