# Supplementary material for: Copy number gains at chr3p25 and chr11p11 are associated with lymph node involvement and survival in muscle-invasive bladder tumors
Source: PLoS One. 2017 Nov 15;12(11):e0187975. doi: 10.1371/journal.pone.0187975 (PMC5687759; doi:10.1371/journal.pone.0187975)
Supplement: S1 Table — (DOCX) [file pone.0187975.s008.docx]

**S1 Table.**

| Gene | Loss/Gain | Tumors in TCGA/COSMIC | Gene Set |
| --- | --- | --- | --- |
| *MTMR14* | Gain | bladder, breast, esophagus, head\|neck, liver, lung, ovary, sarcoma, skin / bone, intestine, pancreas, prostate, stomach | chr3p25 |
| *CPNE9* | Gain | bladder, breast, esophagus, head\|neck, liver, lung, ovary, sarcoma, skin / bone, intestine, pancreas, prostate, stomach |  |
| *BRPF1* | Gain | bladder, breast, esophagus, head\|neck, liver, lung, ovary, sarcoma, skin / bone, intestine, pancreas, prostate, stomach |  |
| *OGG1* | Gain | bladder, breast, esophagus, head\|neck, liver, lung, ovary, sarcoma, skin / bone, intestine, pancreas, prostate, stomach |  |
| *CAMK1* | Gain | adrenal, bladder, brain, breast, cervix, endometrium, esophagus, head\|neck, intestine, kidney, liver, lung, ovary, pancreas, sarcoma, skin, stomach, thyroid / blood\|lymph, bone, prostate |  |
| *TADA3* | Gain | bladder, breast, esophagus, head\|neck, liver, lung, ovary, sarcoma, skin / bone, intestine, pancreas, prostate, stomach |  |
| *ARPC4* | Gain | bladder, breast, esophagus, head\|neck, liver, lung, ovary, sarcoma, skin / bone, intestine, pancreas, prostate, stomach |  |
| *TTLL3* | Gain | bladder, breast, esophagus, head\|neck, liver, lung, ovary, sarcoma, skin / bone, intestine, pancreas, prostate, stomach |  |
| *RPUSD3* | Gain | bladder, brain, breast, esophagus, head\|neck, liver, lung, ovary, sarcoma, skin / bone, intestine, pancreas, prostate, stomach |  |
| *CIDEC* | Gain | bladder, brain, breast, esophagus, head\|neck, liver, lung, ovary, sarcoma, skin / bone, intestine, pancreas, prostate, stomach |  |
| *JAGN1* | Gain | bladder, brain, breast, esophagus, head\|neck, liver, lung, ovary, sarcoma, skin / bone, intestine, pancreas, prostate, stomach |  |
| *IL17RE* | Gain | bladder, blood\|lymph, brain, breast, endometrium, esophagus, head\|neck, kidney, liver, lung, ovary, sarcoma, skin / bone, intestine, prostate, stomach |  |
| *HSD17B12* | Gain | bladder, breast, endometrium, esophagus, head\|neck, kidney, lung, ovary, pancreas, stomach / blood\|lymph, bone, brain, intestine, prostate, skin | chr11p11 |
| *ALKBH3* | Gain | bladder, breast, endometrium, esophagus, head\|neck, lung, ovary, pancreas, stomach / blood\|lymph, bone, brain, intestine, prostate, skin |  |
| *C11orf96* | Gain | n/a |  |
| *ACCSL* | Gain | bladder, breast, esophagus, head\|neck, liver, lung, ovary, sarcoma, skin / bone, intestine, pancreas, prostate, stomach |  |
| *ACCS* | Gain | bladder, breast, esophagus, head\|neck, liver, lung, ovary, sarcoma, skin / bone, intestine, pancreas, prostate, stomach |  |
| *EXT2* | Gain | bladder, breast, endometrium, esophagus, head\|neck, lung, ovary, pancreas, stomach / blood\|lymph, bone, brain, intestine, prostate, skin |  |
| *ALX4* | Gain | bladder, brain, breast, cervix, endometrium, esophagus, head\|neck, kidney, lung, ovary, pancreas, stomach, thyroid / blood\|lymph, bone, intestine, prostate, skin |  |
| *PHF21A* | Gain | bladder, breast, endometrium, head\|neck, kidney, lung, ovary, pancreas, stomach / blood\|lymph, bone, brain, intestine, prostate, skin |  |
| *CREB3L1* | Gain | bladder, breast, endometrium, head\|neck, kidney, lung, ovary, pancreas, stomach / blood\|lymph, bone, brain, intestine, prostate, skin |  |
| *DGKZ* | Gain | bladder, breast, endometrium, head\|neck, lung, ovary, pancreas, stomach / blood\|lymph, bone, esophagus, intestine, lung, ovary, pancreas, prostate, skin |  |
| *EPHA3* | Gain | bladder, brain, breast, cervix, endometrium, esophagus, head\|neck, intestine, kidney, liver, lung, ovary, prostate, sarcoma, skin, stomach / blood\|lymph, bone, pancreas | *EPHA3* |
| *ATE1* | Gain | adrenal gland, bladder, blood\|lymph, brain, breast, cervix, endometrium, esophagus, head\|neck, intestine, kidney, liver, lung, ovary, sarcoma, skin, stomach / bone, pancreas, prostate, thyroid | *ATE1* |
| *HIPK3* | Gain | bladder, breast, esophagus, head\|neck, liver, lung, ovary, sarcoma, skin / bone, intestine, pancreas, prostate, stomach | *HIPK3* |
| *AATF* | Loss | bladder, breast, endometrium, kidney, ovary / blood\|lymph, bone, intestine, pancreas, prostate, skin | *AATF* |
