# Supplementary material for: Copy number gains at chr3p25 and chr11p11 are associated with lymph node involvement and survival in muscle-invasive bladder tumors
Source: PLoS One. 2017 Nov 15;12(11):e0187975. doi: 10.1371/journal.pone.0187975 (PMC5687759; doi:10.1371/journal.pone.0187975)
Supplement: S1 Text — (DOCX) [file pone.0187975.s009.docx]

**S1 Text.**

# Supplemental methods

## Genomic assays

Genomic assays for tumor and normal samples were conducted at the Broad Institute (BI). Copy number (CN) was measured on an Affymetrix Genome-Wide Human Array 6.0 (Affymetrix, Santa Clara, CA) containing 1.8 million markers spanning the entire genome. The BI ran the Affymetrix arrays according to McCarroll et al. (S1), and processed raw (.CEL) files with the Birdseed algorithm (S2) for genotyping and circular binary segmentation (CBS, 25). We re-processed the raw .CEL files with the TumorBoost algorithm (S3) for genotyping and used the Allele-Specific Copy Number Analysis of Tumors (ASCAT) for segmentation (26).

We called CN aberrations (CNAs) for each RefSeq gene (27) in chromosomes 1-22 and X by applying the Genomic Identification of Significant Targets in Cancer 2.0 (GISTIC2.0) algorithm (28). We used the same parameters for the BI and UCSF processing pipelines, including the default removal of hyper-segmented samples (>2500 segments/ sample), joining of neighboring segments based on less than four markers, loss thresholds of log_2_(CN/2)<-0.1 and gain thresholds of log_2_(CN/2)>0.1. The GISTIC2.0 algorithm empirically derives amplification and deletion thresholds. Germline CN variants were removed (list provided with the GISTIC2.0 documentation). If more than one segment overlapped a gene, we used the most extreme CN value to represent that gene.

Expression was measured using the Illumina HiSeq (Illumina, San Diego, CA) platform for mRNA sequencing, with quantification using the RNA-Seq by Expectation Maximization method (S4) and within-sample normalization to a fixed upper quartile. Gene expression levels were median-centered across samples.

Somatic mutations were measured by exome sequencing using the Illumina HiSeq platform and analyzed using the standard Illumina pipeline and BI-developed Picard and Firehose analysis tools.

Methylation was measured using the Illumina Infinium HumanMethylation450 platform, covering 99% of RefSeq genes and 96% of CpG islands from the UCSC database and their flanking regions.

Additional methodological details for the genomic assays and analyses by the BI can be found on the TCGA website (http://cancergenome.nih.gov/).

## Statistical analyses

For our main analyses, the BI-UCSF calls as described in the Methods section and in Supplemental Figure S1 were used. Each gene was coded as a categorical variable indicating whether the BI and UCSF copy number pipelines had 1) a concordant gain call, 2) a concordant loss call, 3) a discordant call, or 4) a concordant non-CNA call (the reference).

In the first analysis stage, we screened all RefSeq genes for CNA associations with LN status using bivariable logistic regression models, keeping genes that had any CNA association (including discordant calls between the BI and UCSF pipelines) with LN positivity at the p<0.1 level. In the second screening stage, we used multivariable elastic net (EN, 30) models to further reduce the number of genes with CNA-LN status associations. We identified the optimal EN model parameters and most predictive subset of genes by using 5-fold cross-validation to reduce prediction error in 100 separate runs, selecting the genes that were the most predictive in 95% of the models. In the third stage of our analysis, we used a sliding window approach to group the genes into “gene sets” if their hg19 positions were within one megabase of neighboring genes, and if the average EN coefficient was within one standard deviation of all EN coefficients with the same sign. In our fourth stage, we conservatively dichotomized gene sets as having gains versus no gains or losses versus no losses, depending on the direction of association in the first two stages.

# Supplemental references

S1. McCarroll SA, Kuruvilla FG, Korn JM, et al. Integrated detection and population-genetic analysis of SNPs and copy number variation. Nat Genet 2008;40:1166–74.

S2. Korn J, Kuruvilla FG, McCarroll SA, et al. Integrated genotype calling and association analysis of SNPs. common copy number polymorphisms and rare CNVs. Nat Genet 2008;40:1253–60.

S3. Bengtsson H, Neuvial P, Speed TP. TumorBoost: Normalization of allele-specific tumor copy numbers from a single pair of tumor-normal genotyping microarrays. BMC Bioinformatics 2010;11:245.

S4. Dempster AP, Laird NM, Rubin DB. Maximum Likelihood from Incomplete Data via the EM Algorithm. J R Stat Soc Series B Stat Methodol. 1977;39(1):1–38.
